# Designing a Self-Management App for Young People With Type 1 Diabetes: Methodological Challenges, Experiences, and Recommendations

**DOI:** 10.2196/mhealth.8137

**Published:** 2017-10-23

**Authors:** Pernille Castensøe-Seidenfaden, Gitte Reventlov Husted, Grete Teilmann, Eva Hommel, Birthe Susanne Olsen, Finn Kensing

**Affiliations:** ^1^ Pediatric and Adolescent Department Nordsjællands Hospital, Hillerød University of Copenhagen Hillerød Denmark; ^2^ Steno Diabetes Center Gentofte Denmark; ^3^ Pediatric and Adolescent Department Herlev Hospital University of Copenhagen Herlev Denmark; ^4^ Department of Computer Science University of Copenhagen Copenhagen Denmark

**Keywords:** adolescents, mHealth, diabetes, chronic condition, self-management, transition, participatory design, usability, feasibility, methodological recommendations

## Abstract

**Background:**

Young people with type 1 diabetes often struggle to self-manage their disease. Mobile health (mHealth) apps show promise in supporting self-management of chronic conditions such as type 1 diabetes. Many health care providers become involved in app development. Unfortunately, limited information is available to guide their selection of appropriate methods, techniques, and tools for a participatory design (PD) project in health care.

**Objective:**

The aim of our study was to develop an mHealth app to support young people in self-managing type 1 diabetes. This paper presents our methodological recommendations based on experiences and reflections from a 2-year research study.

**Methods:**

A mixed methods design was used to identify user needs before designing the app and testing it in a randomized controlled trial. App design was based on qualitative, explorative, interventional, and experimental activities within an overall iterative PD approach. Several techniques and tools were used, including workshops, a mail panel, think-aloud tests, and a feasibility study.

**Results:**

The final mHealth solution was “Young with Diabetes” (YWD). The iterative PD approach supported researchers and designers in understanding the needs of end users (ie, young people, parents, and health care providers) and their assessment of YWD, as well as how to improve app usability and feasibility. It is critical to include all end user groups during all phases of a PD project and to establish a multidisciplinary team to provide the wide range of expertise required to build a usable and useful mHealth app.

**Conclusions:**

Future research is needed to develop and evaluate more efficient PD techniques. Health care providers need guidance on what tools and techniques to choose for which subgroups of users and guidance on how to introduce an app to colleagues to successfully implement an mHealth app in health care organizations. These steps are important for anyone who wants to design an mHealth app for any illness.

## Introduction

Type 1 diabetes mellitus (T1DM) is a major health challenge, particularly among young people, who struggle to manage their condition during the transition from childhood to adulthood. Physical, cognitive, and social changes influence their daily T1DM routines (eg, blood glucose measurement, carbohydrate counting, and insulin adjustment). This frequently results in impaired glycemic control [1], increased risk of acute complications [2], and early onset of long-term complications [3,4].

Parents are important supports for young people to self-manage T1DM [5]. However, parents often report frustrations, stress, and worry regarding their role [6]. Schilling et al [7] define self-management for young people with T1DM as a flexible daily process in which young people and their parents share decision making and responsibility for controlling T1DM. The process whereby young people go from being totally dependent on parents to managing their T1DM by themselves is constantly evolving.

Supporting young people in self-managing T1DM is an integral goal of health care [8]. Unfortunately, self-management support can be complex. Health care providers should both guide insulin management and seek insight into young people’s lived experiences, such as social life, work, and school, to identify challenges affecting self-management. Furthermore, they must pay attention to young people’s needs to develop self-management skills while encouraging and involving parents in supporting their young people [9,10]. Routine care from health care providers appears to have limited effects on self-management and glycemic control [1]. Consequently, supporting young people and parents during the transition from childhood to adulthood is an ongoing challenge for health care providers.

Mobile health (mHealth) apps are promising tools for supporting self-management of a chronic condition such as T1DM [11,12]. They are easily accessible, widely used, and accepted, particularly by young people [13]. They have the potential to improve patient education and enhance communication with health care providers and peers in a convenient and interactive way [14]. Recently, the number of apps to support self-management of chronic conditions such as T1DM in adults has exploded [15]. However, a recent review of mHealth apps for management of chronic physical conditions in adolescents [16] identified only two apps to support adolescents with T1DM [17,18]. Cafazzo et al [17] developed an mHealth app based on interviews with adolescents and their parents to facilitate feedback on blood glucose data. A pilot test (N=20) found an improvement in the frequency of blood glucose monitoring [17]. Frøisland et al [18] tested an mHealth app with a picture-based diabetes diary in addition to a text messaging service in a 3-month pilot study (N=12) and found increased understanding of applied knowledge [18]. Unfortunately, few studies are available, and they are all limited by small sample sizes and the absence of a control group [16]. In addition, mHealth apps are seldom developed on the basis of empirical evidence [19]. In-depth understanding of user needs is often lacking, and the effect of self-management apps is mixed [16,20]. Furthermore, few objective comparisons of methods, tools, and techniques are available to guide health care providers [21] in selecting an appropriate approach for a participatory design (PD) project. Hence, key questions remain unanswered, including which tools and techniques to use and when and where to use them.

This paper presents our methodological recommendations based on experiences and reflections from our 2-year research study. The aim of our study was to develop an mHealth app to support young people in self-managing T1DM.

## Methods

**Figure 1:**
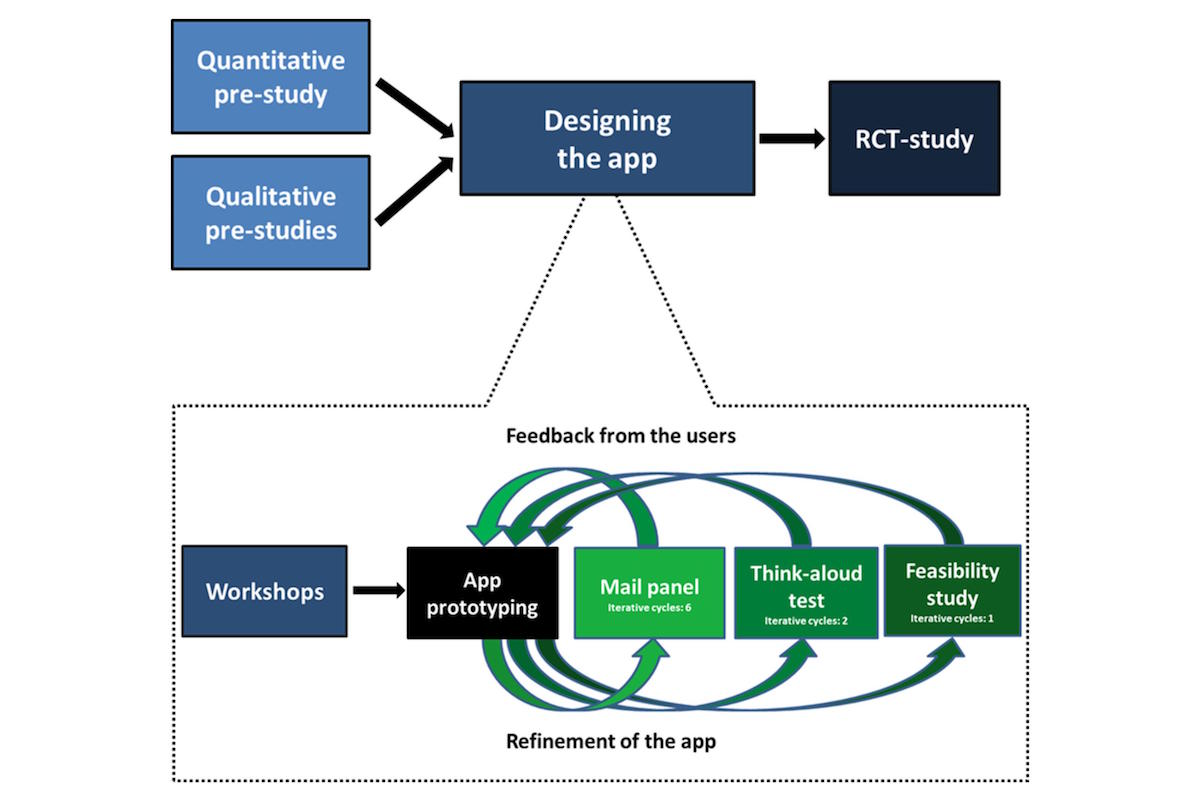
Mixed methods design.

The study used a mixed methods design (Figure 1) comprising (1) quantitative and qualitative pre-studies to identify user needs, (2) quantitative and qualitative studies designing the app, and (3) testing of the effect of app use in a randomized controlled trial (RCT).

The focus of this paper is on the process of designing the app, which was based on qualitative, explorative, interventional, and experimental activities within an overall iterative and PD approach [22] (Figure 1). PD promotes user participation in technology design. It enables designers and end users to learn from each other through understanding each other’s perspectives and priorities [23]. Involving users in developing an intervention is known to result in higher levels of user acceptance and satisfaction [24] and has previously contributed to changes in the design of mHealth interventions [17,18,25]. Several techniques and tools were used to design the app, including workshops, a mail panel, think-aloud tests, and a feasibility study (Figure 1). Throughout the activities, a purposive sampling strategy [26] was used to ensure variation in age, gender, age at onset of T1DM, and location of diabetes care. Table 1 provides a detailed description of the methods, and Table 2 presents participant characteristics.

A steering group was established to ensure a scalable and usable mHealth app. The group consisted of four physician diabetes team leaders, a professor in computer science, a physician and a nurse with expertise in adolescent medicine, the leader of the telemedicine center in the Capital Region of Denmark, and a consultant from the Danish Agency for Digitisation. The steering group met after the pre-studies and after the app was designed to determine if the study phase was complete [22].

The study was approved by the Danish Data Protection Agency (No. 01980 HIH-2012-013, No. 02249 HIH-2013-016, and No. 04015 NOH-2015-031) and performed in accordance with the ethical recommendations of the Helsinki Declaration. Written consent was obtained from participants and from parents if the young people were under the age of 18 years. Confidentiality and anonymity were assured. Ethical approval of retrospective and qualitative studies by Research Ethics Committee is not necessary in Denmark (No. 15000468, Ref. No. H-15013254).

**Table 1:** Detailed description of app design.

Activity	Aim	Participants	Data collection	Data analysis
Workshops	To develop an app to support young people to self-manage T1DM	Inclusion criteria for young people (and parents): 14-22 years old, T1DM ≥1 year, no psychiatric disorders, in pediatric care or adult care Inclusion criteria for health care providers: on the diabetes team at an adult or a pediatric and adolescent diabetes clinic and with ≥1 year experience working with young people with diabetes	Workshop themes: My diabetes; App functions; Sensitive topics; Future; To my parents; Knowledge and skills; Design and language Brainstorming, prioritizing, feedback, and prototyping were recorded and artifacts collected.	Findings summarized for the IT company to describe functionality and modes of interaction.
App prototyping	To design the first version of the app	IT company; young people with T1DM and parents; health care providers; experts (interactive design students, illustrator, journalist, movie creator)	‒	‒
Mail panel	To ensure a reliable and scalable app	See workshop inclusion criteria.	The participants provided written feedback on prototype versions.	Feedback categorized by themes.
Think-aloud test	To ensure a reliable and scalable app	See workshop inclusion criteria. Could not have participated in previous study activities.	Participants “thought aloud” while performing tasks covering the main functions; recordings and observations.	Feedback categorized by themes.
Feasibility study	To test and evaluate the app in a real-life setting	See workshop inclusion criteria; had participated in think-aloud test.	Young people and health care providers tested the app for 5 weeks and completed questionnaires.	Feedback categorized by themes.

**Table 2:** Participant characteristics.

			Workshops (n=41)						Mail panel (n=49)							Think-aloud test (n=16)							Feasibility study (n=44)		
		YP^a^ (n=17)		P^b^ (n=10)		HCP^c^ (n=14)		YP (n=20)			P (n=3)		HCP (n=26)		YP (n=6)		P (n=4)		HCP (n=6)			YP (n=6)		HCP (n=38)	
Age in years, median (SD)			19 (1.7)		‒		‒		19 (2.4)			‒		‒		18 (3.0)		‒		‒		18 (3.0)			‒
Female, n (%)			11 (65)		6 (60)		13 (93)		12(60)			1 (33)		19 (73)		3 (50)		3 (75)		6 (100)		3 (50)			29 (76)
**Pediatric department, n (%)**																										
	Nordsjælland	3 (18)		2 (20)		3 (21)		5 (25)			1 (33)		3 (12)		1 (17)			1 (25)		1 (17)		1 (17)			5 (13)	
	Herlev	2 (12)		3 (30)		1 (7)		2 (10)			1 (33)		4 (15)		1 (17)			1 (25)		1 (17)		1 (17)			8 (21)	
	Roskilde	0 (0)		0 (0)		0 (0)		0 (0)			0 (0)		3 (12)		1 (17)			1 (25)		1 (17)		1 (17)			5 (13)	
**Adult department, n (%)**																										
	Hillerød	8 (47)		4 (40)		4 (29)		8 (40)			1 (33)		6 (23)		1 (17)			1 (25)		1 (17)		1 (17)			6 (16)	
	Steno	4 (24)		1 (10)		6 (43)		5 (25)			0 (0)		8 (31)		1 (17)			0 (0)		1 (17)		1 (17)			9 (24)	
	Køge	0 (0)		0 (0)		0 (0)		0 (0)			0 (0)		2 (8)		1 (17)			0 (0)		1 (17)		1 (17)			5 (13)	
**Profession, n (%)**																										
	Physician	–		–		4 (29)		–			–		13 (50)		–			–		1 (17)		–			15 (40)	
	Nurse	–		–		6 (43)		–			–		9 (35)		–			–		3 (50)		–			17 (45)	
	Dietician	–		–		4 (29)		–			–		4 (15)		–			–		2 (33)		–			6 (16)	

^a^YP: young people

^b^P: parents

^c^HCP: health care providers

### Quantitative and Qualitative Studies Identifying User Needs

Before designing the app, we explored the needs of young people for self-managing T1DM and possible ways to support these needs. We conducted four main activities.

In a retrospective cohort study (n=126) [27], we found that more than 90% of adolescents had a suboptimal level of hemoglobin A1c around transfer from pediatric to adult care. Those who did not attend clinic visits, whose parents were divorced, or who had a learning disability and/or mental health condition had a higher risk of poor metabolic control.

Using visual storytelling with young people with T1DM (N=9) and their parents (N=13) [28], we explored users’ experiences of living with T1DM in individual interviews based on their personal photographs. Young people and their parents experienced the same concerns and challenges related to living with T1DM. They seldom shared these concerns and challenges with each other, which led to misunderstandings, frustration, and conflicts. Four major themes occurred consistently among young people and their parents:

1. Striving for safety. Young people and parents tried to create a “safety net” (ie, hotline, juice, preparing friends) not to risk hypoglycemia. Some adolescents chose to have a high level of blood glucose preventing hypoglycemia, which was supported by some parents.
2. Striving for normality. Young people often felt different from their peers carrying the burden of T1DM. Some tried to be normal by ignoring T1DM, and some parents felt sorry for their child supporting these “breaks.” Peers with diabetes helped many feel normal.
3. Striving for independence. Young people and parents longed for the young people to be independent in T1DM management. However, young people faced obstacles such as lack of T1DM knowledge, skills, and parental support. Some avoided clinical visits to hide their incompetence in self-management.
4. Worrying about the future. Both parties worried about the future, such as the risk of long-term complications. Parents thought their child did not worry and chose not to talk about it. However, young people felt alone with their worries, not sharing them with anyone.

In individual interviews with 24 health care providers (10 physicians, 10 nurses, 4 dietitians; unpublished data), we explored health care provider’s attitudes towards implementing an app in clinical settings. Two major themes were identified: a new way to collaborate and losing control. All health care providers emphasized that an app could help improve their collaboration with the young people by breaking the ice and helping them investigate young people’s real challenges and concerns and by providing ongoing support between clinic visits. On the other hand, health care providers feared losing control of the content of consultations. They feared that their authority would be questioned if they lacked competency with the app and that its use would be too time-consuming. Health care providers preferred electronic messages to be sent to their work email addresses so they would not have to check two devices.

Finally, we identified relevant security regulations to ensure we met the national standards for login procedures and exchanging messages with peers and health care providers. Compliance with security regulations was assured through six regular meetings throughout the design process with consultants from an IT company and from a public health-technology center.

### Designing the App

To develop the mHealth app to support young people in self-managing T1DM, we invited young people with T1DM, their parents, and health care providers to participate in workshops. The first version of the app was developed from the workshop findings.

#### Workshops

Seven workshops were held in November and December 2014. In total, 17 young people aged 16-21 years, 10 parents, and 14 health care providers participated in one or more workshops (Table 2). In addition, 26 individuals with specialized expertise and knowledge, such as dieticians, psychologists, a social worker, interactive design students, a journalist, information technology (IT) consultants, a telemedicine consultant, and other health care providers with an interest in adolescent medicine, participated in workshops to find ways to meet the needs of the young people and their parents. Each workshop included 11-21 participants (Multimedia Appendix 1).

Workshop content was based on the results from our quantitative and qualitative pre-studies [27,28]. The results from the pre-studies were merged by a mixed methods concurrent design [29] to interpret the challenges that young people and parents face living with T1DM. By applying a mixed methods sequential design [29], the results from the pre-studies were used to inform workshops. As an example, visual storytelling elucidated young people’s lack of T1DM knowledge, which was used in Workshop 6 “Knowledge and skills.” Workshop themes are listed in Table 1. Each workshop lasted 2½ hours and included a 5-minute introduction, individual brainstorming, prioritizing ideas, and sketching prototypes based on the ideas. Workshop participants were grouped by whether they were young people, parents, or health care providers. The workshops were audio- and video recorded. All input (Post-it notes, flip charts, prototypes, digital records) were collected and categorized by theme; the IT company incorporated the final list of themes into a description of functionality and modes of interaction.

#### App Prototyping

The IT company built a preliminary version of the app on iOS and Android platforms. Interactive design students were invited to improve the app design and create animations. Young people with T1DM created video self-portraits (“selfies”) on a variety of topics, such as how to tell peers one has T1DM or their experiences with low blood sugar. A professional illustrator created graphic elements, and a journalist wrote youth-friendly texts and tips. Experienced providers on diabetes teams (physicians, nurses, dieticians, psychologists, and social workers) revised and approved the final app content.

We were unable to fulfill all needs identified by users. For example, we did not add a mentor/mentee function due to the complexity of evaluating this intervention. However, a supplementary Web-based secure messaging function for health care providers was developed to enable contact with young people. Multimedia Appendix 1 provides an overview of the needs addressed in each of the workshops, the main ideas identified, and the resulting functionality and modes of interaction in the app.

We aimed to evaluate and refine the app. Our primary concern was its usability and feasibility; the IT company took responsibility for technical testing. Our evaluation relied on three techniques (mail panel, think-aloud testing, and feasibility study). A mixed methods sequential design [29] connected findings and ideas from one qualitative study in the design phase to the next to iteratively inform the prototyping and refinement of the app.

#### Mail Panel

A mail panel comprising 20 young people aged 17-26 years, 3 parents, and 26 health care providers gave feedback on screen shots of the app (mock-ups) and first versions of the app (test flights) in six iterative cycles from March to October 2015. The panel received mail with questions and attached mock-ups (Cycles 1-4) or test flights (Cycle 5-6). The questions focused on layout, content, and functions. The feedback was collected, and the IT company refined the app before the next cycle (Multimedia Appendix 2).

#### Think-Aloud Testing

Think-aloud tests [30] were performed to understand how users experienced the app interface [31]. Six young people aged 15-22 years, 4 parents, and 6 health care providers participated. None of the young people or parents had participated in previous activities. In individual sessions that lasted 15-53 minutes, participants were asked to verbalize their thoughts while performing app-related tasks (Multimedia Appendix 3). Tasks were designed in collaboration with the IT company to test the interface. Participants were prompted if they found thinking aloud challenging (eg, “Tell me what you are thinking”). The sessions were digitally recorded and observed, and comments and nonverbal reactions were noted. Data were categorized by themes. Based on the feedback, the IT company refined the app before next cycle. A total of two iterative cycles were completed (Cycles 5 and 6; Multimedia Appendix 2) in September and October 2015.

#### Feasibility Study

A 5-week feasibility study was conducted from October to December 2015 [32,33]. Author PC-S presented the app to a total of 38 health care providers individually (n=13) or in eight groups (n=25). The presentation included a summary of young people’s and parents’ needs [28], a 15-minute introduction to the app, and two role-playing scenarios in which health care providers were asked to introduce the app and use it as a dialogue ice breaker while a colleague or PC-S played the role of a young person with T1DM. Finally, health care providers were asked to familiarize themselves with the app in their outpatient clinics and use it with at least 2 young people with T1DM during the 5-week study period. Feasibility was evaluated by an electronic questionnaire sent to health care providers that contained questions such as:

- Does the app work on your device?
- To whom have you introduced the app?
- What challenges have you experienced using the app?
- What has to be changed before conducting an RCT evaluating the app?

Six young people aged 15-22 years who had participated in think-aloud tests were individually introduced to the app in 15-minute sessions and asked to test it for 5 weeks at home and in cooperation with their health care providers and parents. They received a paper questionnaire with questions such as:

- Has the app helped you?
- How has the app helped you?
- Have you used the app in collaboration with health care provider or parent?
- Would you recommend the app to peers?

The questions were based on previous experiences of the IT company with app development; 2 health care providers and 2 young people assessed face validity before the questionnaire was distributed to test participants. Their responses were categorized into themes, and the IT company made final refinements of the app based on the feedback.

#### Young With Diabetes App

**Figure 2:**
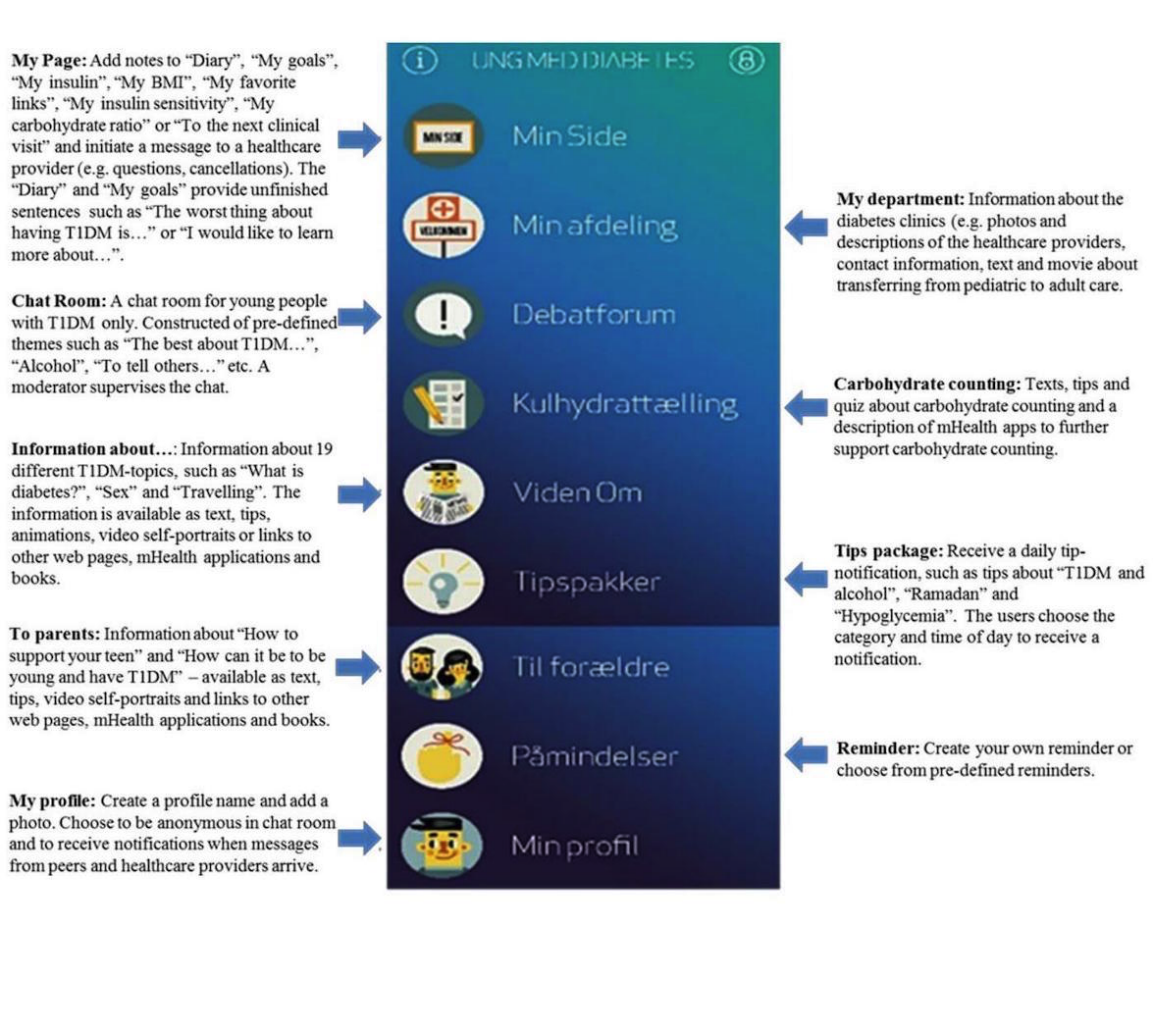
Young with Diabetes.

The final mHealth solution consists of the app “Young with Diabetes” (YWD) and an additional Web-based mail module through which health care providers receive messages from young people. The eight main functions in the app are outlined in Figure 2.

## Results

Our experience yielded valuable learnings and a set of recommendations for future app development (Table 3).

### Mixed Methods Design

A mixed methods design allowed us to obtain a nuanced understanding of users’ needs and challenges before the workshops [29]. This knowledge was essential to guiding both workshop content and design of the app. In addition, we used a variety of qualitative techniques to explore how users viewed and assessed YWD. This gave us a better understanding of the app content, user interface, and technical issues and was invaluable to further refining the app before implementation. By applying a mixed methods design [29], the findings from the pre-studies informed the design process resulting in a final version of YWD that met the users’ needs and challenges. As an example, one of the findings from visual storytelling was “striving for normality.” Young people strove for normality in order to not feel different from their peers without T1DM. In addition, meeting peers with T1DM often helped them feel normal. This finding was approached in Workshop 3 “Sensitive topics,” where ideas were generated on how to support young people to feel normal. The final app consequently ended up with a chat room and video self-portraits to share experiences with peers, in addition to an information topic on how to tell others that you have T1DM. Furthermore, the parent section informed parents on how it can actually be to have T1DM including the young peoples’ struggle for normality.

Applying a mixed methods design is consistent with a recent design study [34] reporting that qualitative and quantitative results contributed to a comprehensive understanding of the technology and area of concern. On the contrary, a recent PD project [35] developed a patient-centered mHealth app for young people with T1DM and their parents using only qualitative methods, which provided limited information for improving the app. A mixed methods design is highly recommended for future PD projects to gain a comprehensive and nuanced knowledge of the area of concern and meet the needs of users [29].

### Participatory Approach

Our PD approach engaged all types of end users (young people, parents, and health care providers) in designing the app. End users were essential collaborators, helping researchers and designers further understand their challenges and needs, generating ideas, giving feedback, and testing the new technology to ensure a usable and feasible product. Similar to previous PD projects [17,18,25], user input contributed to crucial changes in the technology. We found that different types of users had different approaches when engaged in workshop activities. For example, young people generally found it easier to create paper prototypes than did parents, and health care providers and young people often shared new apps with each other, fostering new ideas. We also found it useful to separate workshop participants by user type because they seemed to share ideas more readily than they did in mixed groups. More research is needed to improve our understanding of how best to engage subgroups of participants and which tools and techniques best suit each type of end user.

Giving a voice to end users and designers and respecting their different views is key to success when developing new technology [17,18,24,25]. However, we found it challenging to resolve conflicting views on functionality while taking resources, such as finances and time, into account [21,36]. The seven workshops yielded large amounts of user input and feedback: 46 hours of digital records in addition to several Post-it notes, flip charts, pictures, etc. PC-S, the IT company, and the steering group made final decisions about app content. Taking resources into consideration, they had to eliminate some user-requested functions, such as a mentor/mentee function, a monthly newsfeed, customizing the app, and the ongoing possibility of uploading new video self-portraits. Future app development should consider incorporating these functions, since customization is especially known to be critical for app engagement [35]. In addition, guidelines are needed as to how to manage (collect, analyze, and prioritize) data in future PD projects [36,37]. For example, we were challenged by questions such as: Should digital records be transcribed? How should data be analyzed in PD activities? How do we prioritize and eliminate ideas that are highly valued? Such guidance may help to limit the large amount of data that often challenge PD projects [36] and reduce time-consuming data management activities.

### Diverse Team of Experts

Researchers, designers, or end users alone could not have created YWD. The expertise of a diverse team was crucial to building a new health care technology platform and creating the content (eg, information, quizzes, pictures, illustrations, movies, video self-portraits). The authors collaborated with educational institutions such as the Danish School of Media and Journalism to obtain needed expertise at minimal cost and provide students with “real life” projects. In addition, a public-private partnership was established between the hospital and the IT company. The partnership created a team of highly engaged stakeholders who contributed to the study and shared an interest in the study’s success. We strongly recommend establishing a multidisciplinary team before developing an mHealth app in health care, which is consistent with previous studies [38]. In addition, it may be beneficial for health care providers, who are often inexperienced in designing new technology, to cooperate with an expert in conducting PD projects to guide the design process. Stakeholders who are considering developing an mHealth app may benefit by meeting with experts, such as innovation and security consultants and lawyers (to draft a partnership agreement) very early in the process. For instance, a group has been established in the capital region of Denmark to guide health care providers through facets of new app studies such as addressing security regulations, facilitating workshops, etc [39].

**Table 3:** Recommendations for future app development.

Challenges	Experiences	Recommendations	Suggestions for further research
Engaging user subgroups	Participants who are separated in user groups seemed freer to share ideas than they may have been in mixed groups.	Separate participants in user groups in workshops sessions.	What tools and techniques are best suited for different types of end users (ie, young people, parents, health care providers)?
	Young people found it easier to create paper prototypes, compared to parents and health care providers.	Creating paper prototypes is an effective tool for engagement, especially with young people in activities to generate ideas.	
Resolving conflicting views on functionality	The IT company and the steering group made the final decisions about the content of the app.		Incorporate eliminated functions in a future version since users wanted them.
	Functions (mentor/mentee-function, customization, and monthly newsfeed) were eliminated due to lack of resources.		How to prioritize and eliminate user ideas?
Meeting requirements for building an mHealth app	A diverse team of experts was crucial to meeting the challenge of building a new technology platform within health care.	Invite end users, designers, and a diverse team of experts (eg, illustrators, journalists) to participate in workshops.	
		Collaborate with other educational institutions to meet the need for expertise with minimal cost.	
		Establish public-private partnerships to combine resources and ensure engagement from all stakeholders.	
		Consider engaging an innovation consultant to guide the PD process.	
		Set aside enough time to build the app – it always takes more time than expected.	
		Invite users to participate in meetings with the IT company during app building.	
Designing and refining technology in a rapid, low-cost way	Main activities resulted in large amounts of data (eg, 46 hours of digital records from workshops).	Prolong the prototype stage before developing complex expensive technology.	Guidelines are needed on how to collect, analyze, and prioritize data.
	Ongoing user input from iterative cycles helped designers understand user needs and refine the app.	Consider workshops as an ongoing iterative activity in which users give feedback and propose new ideas to prototypes.	More efficient methods, tools, and techniques are needed to meet the rapid development within technology to avoid outdated app versions.
	Expensive technology challenged our ability to meet the users input.	Use living labs to simulate hospital or home settings to try out paper prototypes and explore future ways to use the new technology.	How do we reduce resource (money and time) use?
Improving the user interface	The mail panel functioned as a consulting panel and provided feedback in a short time that improved the app content. The think-aloud tests explored how users assessed the app (ie, navigation, technical errors).	We highly recommend both a mail panel and think-aloud tests in future PD studies, given the valuable input and the low cost and speed of conducting these techniques.	Solicit larger panels using social media (eg, Facebook, Twitter) to comment and share ideas.
	Combining mail panel and think-aloud tests resulted in a substantial reduction of user problems.	Add digital videos and screen records in think-aloud tests to register physical actions, supporting the interpretation of the results.	Introduce a panel in an earlier phase to supplement or replace face-to-face workshops.
Implementing technology in health care	Interviews with health care providers helped us understand barriers to introducing new technology.	Include end users in all phases of a PD project to ensure adoption.	How to teach health care providers to use new technology in collaboration with young people and parents?
	Workshops, mail panel, think-aloud tests, and feasibility study helped us to ensure a user-friendly app.	Feasibility test new technology prior to implementation.	
	The feasibility study revealed implementation barriers.	Provide a hotline in case of technical difficulties.	
		Teach health care providers how to use the technology prior to test.	

### Iterations

Iterative cycles were introduced based on the feedback from the mail panel, think-aloud tests, and the feasibility study. However, the expensive nature of the technology, which had been developed after workshops before the iterative cycles began, challenged our ability to address user input that arose during iterative cycles after app development. Future studies should prolong the prototype stage. Workshops could be considered as an ongoing iterative activity to enable users to give feedback on mock-ups and propose new ideas. In addition, living labs [40] and scenarios could be used to simulate a hospital or home setting where users could explore paper prototypes early in the design process, reflecting on ways to use the new technology [23]. Living labs have previously proved useful to making quick adjustments to prototypes [41] before developing complex, expensive technology [23]. Currently, a need exists to evaluate new approaches and explore more efficient methods, tools, and techniques for rapid technology development.

### Mail Panel and Think-Aloud Tests

YWD was evaluated by a mail panel and think-aloud tests before the feasibility test. The mail panel provided valuable user feedback in a short time frame, such as when researcher and designers were uncertain about the front-page design of YWD (Multimedia Appendix 2). The panel primarily improved the app content by reporting incorrect or missing information, misspellings, and information overload, whereas the think-aloud tests largely explored how users assessed the app interface (navigation, technical errors, and layout). As an example, think-aloud test participants were not able to locate the “tips package” located in the reminder function. This resulted in the creation of a separate tips package function. Similarly, think-aloud test participants perceived the icon illustrating the carbohydrate-counting quiz score as a download symbol and began to wait; the icon was subsequently changed. Combining the mail panel and think-aloud tests made it possible to improve the app’s usability [36]. In keeping with previous usability studies, we found a substantial reduction in user-identified problems between iterations [21]. Given the valuable input and the low cost and speed with which these techniques can be used, we highly recommend both mail panel and think-aloud tests in future PD projects. In think-aloud tests, we recommend adding digital videos and screen recordings to register physical actions, making the analyses more objective [21] and supporting designers’ interpretation of the results. Technology evaluations may be expanded by engaging a larger panel via social media such as Facebook and Twitter, thus disseminating ideas in a viral way and accomplishing iterations quickly [42]. Finally, it could be interesting to introduce a panel in an earlier study phase to supplement or replace face-to-face workshops in the process of generating new ideas. PD practitioners are exploring these possibilities to facilitate participation and further adoption of new technology [23].

### Feasibility Testing

Finally, the 5-week feasibility study evaluated YWD use in real-life settings. Young people’s and health care providers’ attitudes towards the app were explored, revealing practical and technological challenges. These challenges would otherwise have been apparent only after implementation. Young people found the app informative and found that it provided them with a range of self-management support, such as the opportunity to write to their health care providers. They all reported that they would recommend the app to peers. In addition, health care providers described the app as both intuitive to use and relevant to collaborating with young people with T1DM. However, some technical difficulties were reported regarding screen setup and unstable wireless access networks at diabetes clinics, and sending or receiving messages did not always work. None of the young people initiated messages to peers or created notes. To get more activity in the “Chat Room,” young people suggested more participants, notifications about new messages, and input from a moderator. Testing YWD’s feasibility in a “real” clinical setting aligns with Medical Research Council guidance for the evaluation of complex interventions [43]. We found the feasibility study to be invaluable to further adaptation of YWD; we improved wireless access at clinics, addressed technical issues, and introduced message notifications. We hope that feasibility testing will help prevent challenges and frustrations that often follow the introduction of new health care technology.

### Implementation in Real-Life Settings

To facilitate successful implementation, we explored health care providers’ perspectives during interviews before designing the app; addressing their concerns was “the first step in embedding the system into practice” (p. 573 [25]). However, during the feasibility study, only half of the providers (n=19) introduced the app to at least one person (young people, colleague, or family). Of these, 14 providers successfully introduced the app to one or more young people. Reasons that health care providers did not introduce the app included a lack of eligible patients, limited time, unstable wireless access, or forgetting to do so. To ensure that providers were able to use the app, we asked them to rate themselves on a readiness scale from 1-10, with 1 representing not ready and 10 representing absolutely ready [44]. Nearly half (16/38, 42%) of providers rated themselves lower than 7 (median 7, range 1-10). This is consistent with previous studies documenting challenges in adoption of new health care technology [45]. Several explanations should be considered. First, the introduction of new technology may cause a disruptive change in providers’ usual workflow [46] and the benefits of the technology may take time to materialize [47]. Our interviews with health care providers (unpublished) revealed that some feared that their authority would be questioned if they were not fully competent at using the app. The training session included “hands-on” activities to simulate real-time scenarios, as recommended [48,49]. However, we may not have spent enough time practicing the scenarios or could have used another teaching approach. In addition, lack of competency at app use may have influenced health care providers’ use of YWD, as identified in other studies [50,51], making it challenging for them to engage effectively with young people via the app. Furthermore, some providers were challenged by technical issues related to wireless access and the app itself. Finally, the extensive supporting material (23 informational articles, 43 video selfies, 3 videos of the adult department, 4 animations, several tips packages) required health care providers to spend many hours becoming familiar with YWD. Time should be allocated for health care providers to become familiar with a new technology before introducing it into their practice.

Future implementation of YWD will require regular updates and ongoing staff training to address challenges. For instance, some providers may need more training due to lack of eHealth skills [52,53]. In addition, there is a need for continuous app support via a hotline to overcome technical challenges and wireless access barriers [54,55] and to provide a smooth transition to a new workflow and success in actual usage [50,56]. Health care providers often function as gatekeepers deciding which patients they believe the technology will work for [57]. It could be interesting to consider mHealth apps as “prescriptions” in the future, since prescriptions are often seen as more “serious” recommendations, hopefully enhancing the use of technology. Our feasibility study was followed by health care providers spending more time studying the app, additional training in introducing the app to young people, an app refresher session, help reinstalling the app, frequent app support visits from PC-S, and establishment of a hotline for technical support. Following these additional interventions, all providers rated themselves at 7 or higher on the readiness scale.

## Discussion

### Strengths and Limitations

The strengths of our study are the mixed methods design and rigorous PD approach applied to ensure that the app would be relevant to all groups of end users and the evaluation of YWD in real-life settings [58]. However, time and financial resources limited our ability to fulfill all user needs. We invited users to participate in more than one activity, which may have biased our results toward favoring their preferences and perhaps made it more difficult for them to continue to think critically about the study. To mitigate this risk, we invited a new set of end users to participate in think-aloud tests and feasibility studies. However, one may argue that participation in more than one main activity is required to gain a mutual understanding of the design process. Due to limited time and economic resources, no parents were included in the feasibility study, thus we do not know if the app is feasible or suitable for them. This shortcoming should be approached by including parents in future tests of YWD. We were unaware of the relevance of including users in the initial meetings with the IT company during the design of the first versions of YWD. We did not use validated questionnaires to score a wider variety of concepts related to eHealth literacy [59], such as functionality, modes of interaction, and user acceptance [60] since these questionnaires were not available in Danish at the time. Doing so would be preferable in future studies. We tested the app over a relatively short period of time and thus do not know its long-term impact or the likelihood that users will stop using it over time [61,62]. Finally, PC-S performed all the main activities. This may have influenced participants’ reflections during think-aloud tests and their evaluation of the feasibility study, affecting the resulting mHealth solution in unknown ways.

### Conclusion

Our study is an important step for any stakeholder who wants to design an mHealth app for any illness. It paves the way for future PD projects within health care by underscoring the importance of including end user groups during all phases and establishing a multidisciplinary team to provide the wide range of expertise required to build an mHealth app. Before building expensive app versions, we suggest prolonging the prototype stage through workshops, mail panel, think-aloud tests, or scenarios in living labs. Our study highlights a crucial need to develop and validate more efficient PD tools and techniques and to focus on the tools and techniques that are best for specific user groups. Finally, we need to understand how to successfully and efficiently introduce an app to health care providers before we can succeed in implementing mHealth apps in real-life health care settings. An RCT is currently examining the efficacy of YWD in improving the self-management skills of young people with T1DM by measuring hemoglobin A1c and three psychometric scales: Perceived Competence in Diabetes Scale [63], Health Care Climate Questionnaire [63], and Problem Areas in Diabetes care survey [64]. The RCT is followed up by individual interviews qualitatively evaluating YWD. If it proves effective, YWD may potentially serve as a model for supporting self-management and transitions for young people with other chronic or long-term conditions.

## Appendix

Multimedia Appendix 1 Workshop overview.

Multimedia Appendix 2 Description of the iterative cycles.

Multimedia Appendix 3 Think-aloud tasks.

## Abbreviations

IT: information technology
PD: participatory design
RCT: randomized controlled trial
T1DM: type 1 diabetes
YWD: Young with Diabetes app
