# Supplementary material for: Designing a Self-Management App for Young People With Type 1 Diabetes: Methodological Challenges, Experiences, and Recommendations
Source: JMIR Mhealth Uhealth. 2017 Oct 23;5(10):e124. doi: 10.2196/mhealth.8137 (PMC5673883; doi:10.2196/mhealth.8137)
Supplement: Multimedia Appendix 1 [file mhealth_v5i10e124_app1.pdf]

Multimedia Appendix 1. Workshop overview

Workshop	Participants	Needs and possibilities approached	Main ideas identified	Functionality and modes of interaction - based on workshop ideas
**Workshop 1: My Diabetes**	**Young people (7), Parents (2), Health care providers (2), Persons with special expertise and knowledge (3)**	**Retrospective study**<br>• A general need to improve glycemic control<br>• Support those not attending clinic visits<br>**Interviews with young people and parents**<br>• Support young people and parents to share concerns and challenges.<br>• Talk about and identify concerns, challenges and strategies in regard to hypoglycemia, worries about future, feeling different and incompetent in T1DM management.<br>**Interviews with health care providers**<br>• Messages from young people should be sent to health care providers' work mail.<br>• The app could be a potential icebreaker to elucidate challenges and concerns.<br>• A need for ongoing support	**Ice-breaker**<br>• What do you want to tell us?<br>○ HEADS<br>○ This is me… (look at the "whole" person)<br>○ Support from surroundings? What support do you need?<br>○ How openly do you speak about your T1DM?<br>○ How much do you want your friends to know about diabetes?<br>○ What is most important for you right now?<br>○ Where do you see yourself and your diabetes in 5 years?<br>○ The biggest challenge about T1DM?<br>○ Positive/negative experiences<br>○ What do you want us to know?<br>• How do you want to tell us? (Pictures, Unfinished sentences, Diary, Agenda for the consultation)<br>**Other ideas**<br>• My measures, pump, insulin, goals, next consultation, ordering equipment, favorite web pages	**Main-function**<br>○ "My Page"<br>**Functions, modes and interactions:**<br>Contact your provider/notebook<br>○ Unfinished sentences/questions<br>○ Add photos<br>○ Next consultation<br>○ Diary (post a photo/text)<br>○ My measures, pump, insulin, goals, favorite web pages
**Workshop 2: The app's functions**	**Young people (4), Parents (3), Health care providers (2), Persons with special expertise and knowledge (2)**	**Retrospective study**<br>• A general need to improve glycemic control<br>• Support those not attending clinical visits<br>**Interviews with young people and parents**<br>• Support young people and parents to share concerns and challenges.<br>• Support parents to help them support their young people.<br>• Talk about and identify concerns, challenges and strategies in regard to hypoglycemia, worries about future, feeling different and incompetent in T1DM-management.<br>**Interviews with health care providers**<br>• The moderator role cannot be provided by health care providers<br>• Messages from young people should be sent to health care providers' work mail.<br>• The app could be a potential icebreaker to elucidate challenges and concerns.<br>• A need for ongoing support<br>**Identified and met the security regulations**<br>• Contact function fulfills security regulations<br>• Chat room fulfills security regulations	**Reminder function**<br>• Set a reminder (predefined, define your own)<br>• How to get a reminder (repeat, notification, share with parents)<br>• Sync with calendar<br>**Contact-function**<br>• Contact info: provider's picture, name, phone number, hours available<br>• Share blood glucose measures/cancel/book appointments/set the agenda<br>• Info about answering time<br>• Message notification<br>**Chat room**<br>• Profile (picture, gender, age, department, be anonymous)<br>• Predefined groups (party/alcohol, food, sport)<br>• Open/private rooms<br>• Find someone with similar interest<br>• Share good and bad experiences/share knowledge/ask peers/upload tips/help to accept T1DM/ post an invitation<br>• For young people only<br>**Mentor**<br>• Find/write a mentor<br>• Apply to be mentor<br>• Private room, mentor/mentee<br>• To help accept diabetes/To gain knowledge<br>• Mentor supervision	**Main function**<br>○ Reminder<br>○ Chat room<br>○ "My Page"<br>**Functions, modes and interactions:**<br>Reminder<br>○ Predefined/define your own reminders<br>○ Set reminder interval<br>○ Notification<br>Chat with peers<br>○ Create a profile<br>○ Predefined groups<br>○ Create a message/ post a comment or invitation<br>○ Open chat rooms<br>○ Receive a message notification<br>Mentor<br>○ Create a mentor profile<br>○ Contact mentor<br>○ Reply to be mentor/to get a mentor<br>○ Mentor supervision<br>Contact your provider
**Workshop 3: The sensitive topics**	**Young people (3), Parents (2), Health care providers (3), Persons with special expertise and knowledge (17)**	**Retrospective study**<br>• A general need to improve glycemic control<br>**Interviews with young people and parents**<br>• Support young people and parents to share concerns and challenges.<br>• Support parents to help them support their young people.	**Methods to identify the real challenge ("icebreaker")**<br>○ Contact your health care provider (share diary/share unfinished sentences/post a picture/set the agenda)<br>○ Video self-portraits<br>**Information about "the soft themes…"**<br>○ Information about… (text, tips, video self-portraits, links)<br>**Others' experiences (sharing knowledge and experiences)**	**Main functions**<br>○ "My Page"<br>○ Chat room<br>○ Information-section<br>○ Tips-packages<br>○ To parents<br>**Functions, modes and interactions:**

		• Talk about and identify concerns, challenges and strategies in regard to hypoglycemia, worries about future, feeling different and incompetent in T1DM management.<br>**Interviews with health care providers**<br>• The app could be a potential ice-breaker to elucidate the challenges and concerns.<br>• A need for ongoing support	o Peer stories<br>o Events<br>o Mentor<br>o Interactive diary/blog (with gate-keeper)	Contact your provider/notebook<br>o Diary/Unfinished sentences/Post a picture<br>Chat with peers<br>o Pre-defined groups (feeling different, best/worst thing about diabetes, fuck diabetes)<br>Information (Text, links, tips, video self-portraits, animations)<br>o my body/diabetes stress/feeling different/hypo<br>Tips-notifications<br>o Receive a tip-notification about (my body, diabetes stress, feeling different, hypo)<br>To parents (text, links, tips, video self-portraits)<br>o what challenges can young with diabetes face/how to support
**Workshop 4: Future**	**Young people (5), Parents (3), Health care providers (3), Persons with special expertise and knowledge (1)**	**Retrospective study**<br>• A general need to improve glycemic control<br>**Interviews with young people and parents**<br>• Support young people and parents to share concerns and challenges.<br>• Support parents to help them support their young people.<br>• Talk about and identify worries about future.<br>**Interviews with health care providers**<br>• The app could be a potential icebreaker to elucidate the challenges and concerns.	**Information about…**<br>o Pregnancy/T1DM complications/travel/education/family, alcohol/research update<br>o Support parents to get an overview, how can it be to be a parent.<br>Sharing experiences<br>o Mentor<br>o Chat<br>o Positive stories<br>o Video self-portraits<br>o Contact your provider<br>Youth consultations<br>o Give space to difficult thoughts<br>o Talk about potential challenges (worries about future)<br>o What is actually challenging<br>o Treatment should fit our lives, not the opposite<br>o Praise and be positive<br>o Motivate, ask<br>o App and patient at the same level/address problems earlier<br>o Personal consultations – what do you want to talk about<br>**My adult department**<br>o Movie (how was it to move to adult department, what can adult department offer/who are we)<br>o Transfer information<br>o My new provider team (to be able to contact new provider, work experience, picture)	**Main functions**<br>o "My Page"<br>o "My department"<br>o Chat room<br>o Information section<br>o Tip packages<br>o To parents<br>**Functions, modes and interactions:**<br>Contact your provider/notebook<br>o Diary/unfinished sentences/post a picture/set the agenda<br>My department (text/movies/photos of providers)<br>o Information (welcome, transfer to adult care, contact)<br>Chat with peers<br>o Predefined groups (future, pregnancy, travel)<br>Information (text, links, tips, video self-portraits, animations)<br>o Late complications/pregnancy/travel/sport/ what to tell others (e.g., at work)<br>Tips notifications<br>o Late term complications/pregnancy/travel/sport<br>To parents (text, links, tips, video self-portraits)<br>o Challenges the app can address/how to support
**Workshop 5: To my parents**	**Young people (3), Parents (3), Health care providers (2), Persons with special expertise and knowledge (5)**	**Retrospective study**<br>• A general need to improve glycemic control<br>• Families with divorced parents need extra support<br>**Interviews with young people and parents**<br>• Support young people and parents to share concerns and challenges.<br>• Support parents to help them support their young people.<br>• Talk about and identify the parties' concerns, challenges and strategies in regard to hypoglycemia, worries about future, feeling different and incompetent in T1DM management.<br>**Interviews with health care providers**<br>• A need for ongoing support	**A main function "To parents" (facts and information/chat, link to Facebook/tools (conflicts, anxiety)/video self-portraits)**<br>Information about being young with diabetes<br>o Brain development/What can be challenging (feeling different, "creating space" to measure blood glucose before everyone else runs out to school break)/normal reaction/information about alcohol, turning 18, diabetes etc. – provided with the basic knowledge to support.<br>Information about being a parent to a young person with diabetes<br>o Acknowledge the challenge. It is normal to be afraid/frustrated/You know your teen the best. Keep support/partner relationship – can be a challenge<br>Information about how young people wants to be supported<br>o Ask what kind of support <u>we</u> need/be available when we need you/talk about how we are doing (not only measurements)/show interest/help us during the difficult	**Main functions**<br>o "My department"<br>o Information section<br>o Tips packages<br>o To parents<br>o Reminder<br>**Functions, modes and interactions:**<br>My department (What happens when my teen turns 18 and moves to adult department?)<br>o Information (welcome, transfer, contact, can parents join consultations?)<br>o Movies<br>o Pictures of providers<br>Information ssection (Text, links, tips, video self-portraits, animations)<br>o Information about T1DM and …

			days/listen to our frustrations/and stand by our side when we are not able to meet the expectations/do not hide/avoid diabetes – we then feel alone/don't be too controlling. We do not want to be interviewed by our parents. It makes us feel even more different/focus on realistic goals/support healthy lifestyle, education etc./do not seem like you know more about diabetes than us/do not interrupt when someone asks about our diabetes<br>○ Parent tools (conflict management/dialogue tools/how to let go)<br>Peer experiences<br>○ Parent to parent chat/meetings/link to face book<br>○ Young people present/tell their experiences (video self-portraits)	Tips notifications<br>○ Tips about T1DM and…<br>○ About being young with diabetes<br>○ How to support your teen<br>To parents (text, links, tips, video self-portraits)<br>○ Challenges faced by young with T1DM<br>○ How to support<br>○ What happens when my teen turns 18?<br>○ Siblings and relationship<br>Reminder
**Workshop 6: Knowledge and skills**	**Young people (6), Parents (4), Health care providers (6), Persons with special expertise and knowledge (2)**	**Retrospective study**<br>• A general need to improve glycemic control<br>• Families with divorced parents need extra support<br>**Interviews with young people and parents**<br>• Support parents to help them support their young people.<br>• Talk about and identify concerns, challenges and strategies in regard to hypoglycemia and being incompetent in T1DM management.<br>**Interviews with health care providers**<br>• A need for ongoing support	**How to receive information?**<br>○ Text/animation/alcohol list/video self-portraits/links/tips<br>○ Seek information - box<br>**Information about…**<br>○ Turning 18: What support can I get from community before/after turning 18/My social rights/What should I do when I turn 18?<br>○ Drugs: Don't/how it influences blood glucose level/diabetes/consequences/where to get help/why drugs are dangerous?<br>○ Drivers' license: What does it take to get a drivers' license (blood glucose level, insulin shock, complications)/renewal of drivers' license/tips – before driving/getting low during driving etc.<br>○ Sex: How sex influences your blood glucose/how to prevent being low/"lines" – how to tell your partner about diabetes/prevention/pump – keep it on/or not/something not possible<br>○ Sport: How does sport influence my body/help my diabetes/protein supplement and diabetes/food before and after exercise/insulin dosing tips/tips before, during, after exercise /when to measure blood glucose (before, during, after)/how to keep a stable blood glucose level during sport/how do different types of sport influence my blood glucose<br>○ Alcohol**:** how alcohol influences my body/blood glucose/list of carbs in alcohol/pump settings and alcohol/info to friends (drunk vs. having hypoglycemia)/what is best to drink<br>○ Travel**:** SOS, what do I do when losing insulin/break down of pump/how to store insulin/different time zones/what to remember – list/different food influences on blood glucose/from pump to pen/pump passport/how does heat/cold influence blood glucose/change in physical activity)<br>○ Hypoglycemia**:** What to eat?/how should I prepare friends?/glucagon/prevent hypoglycemia when exercising/symptoms/definition/what happens?/hotline)<br>○ Ketoacidosis**:** what is it/symptoms/why does it happen?/can I measure it?/what should I do?)<br>○ Pump/pen**:** Accessories/note insulin dosing/how to convert pump to pen/how and where to inject/SOS: acute pump failure/how to calculate insulin sensitivity/insulin nodes/type of insulin/ump pros/cons)<br>○ Food**:** List of carbohydrates in food/link to other apps/fast	**Main functions**<br>○ "My page"<br>○ Carbohydrate counting<br>○ Chat room<br>○ Information section<br>○ Tips packages<br>**Functions, modes and interactions:**<br>"My page"<br>○ Ask a question of your health care provider<br>○ Set the agenda<br>○ Note "My insulin"<br>○ Calculate and note "Insulin sensitivity"<br>Carbohydrate counting<br>○ Information/tips/how to count/quiz/description and links to other carbohydrate-counting apps<br>Chat room<br>○ Ask peers (predefined groups of different diabetes sections)<br>Information (Text, links, tips, video self-portraits, animations)<br>Tips notifications

			and slow carbs/insulin dosing/food to keep blood glucose level stable/what does a dietician offer?/food and sport/protein supplement/how to avoid gaining weight/simple recipes/when does my blood glucose rise after different food intake/how to count carbohydrates<br>**Carbohydrate counting, main function**<br>○ Introduction to carbohydrate counting apps/food and sport/insulin dosing/social (chat/ask your dietician/blog)/party (alcohol lexicon, eat before sleep)/tips/fast and slow carbs/hypoglycemia – what can I eat?	
**Workshop 7: Design, Language and Message**	**Young people (5), Parents (1), Health care providers (0), Persons with special expertise and knowledge (15)**	**Interviews with young people and parents**<br>• Support young people and parents to share concerns and challenges.<br>• Support parents to help them support their young people.<br>• Talk about and identify concerns, challenges and strategies in regard to hypoglycemia, worries about future, feeling different and incompetent in T1DM management.<br>**Interviews with health care providers**<br>• The app could be a potential icebreaker to elucidate the challenges and concerns.	Layout<br>○ Simple/like customizing/blue and green colors with icons/should not remind about diabetes/funny/Main functions should be distinct/sub-menus at the side of the screen/icons instead of text/no color-box pictures<br>○ Article layout (heading, short summary, detailed description – all articles should be designed the same way, tips/good ideas)<br>Navigation<br>○ Easy to navigate/main categories with submenus<br>Content<br>○ No log book – too much work<br>○ Place “Seek information” function at the top of the screen<br>○ Tips/articles/chat<br>Language<br>○ Neutral language - not threatening. (e.g., be careful about writing “this is dangerous…” – instead “take precautions”)<br>Message<br>○ Focus on possibilities/positive things instead of limitations/negative things	**Layout**<br>○ Blue and green colors/icons with illustrations/main functions distinct/submenus at the side of the screen<br>○ Article layout (heading, short summary, detailed description, including tips, possible to choose to see video)<br>**Navigation**<br>○ Easy to navigate/main categories with sub-menus<br>**Content**<br>○ “My page” (including the unfinished sentence “The best about diabetes…”)/articles/chat (including the pre-defined group: “the best about diabetes”)/<br>○ “Seek information” function in the top of information section<br>**Language**<br>○ Neutral – not threatening<br>**Message**<br>○ Positive/focus on possibilities
