# Supplementary material for: Designing a Self-Management App for Young People With Type 1 Diabetes: Methodological Challenges, Experiences, and Recommendations
Source: JMIR Mhealth Uhealth. 2017 Oct 23;5(10):e124. doi: 10.2196/mhealth.8137 (PMC5673883; doi:10.2196/mhealth.8137)
Supplement: Multimedia Appendix 2 [file mhealth_v5i10e124_app2.pdf]

Multimedia Appendix 2. Description of the iterative cycles

Questions	Participants	Usability issues		Resulting changes
**Cycle 1, mail panel**				
Mock-up 1-3 is one proposal of the look of the app. Mock-up 4-5 is another proposal. What do you think about the design (colors, illustrations, icons)? What do you prefer?<br>***Mock-up 1-3***<br>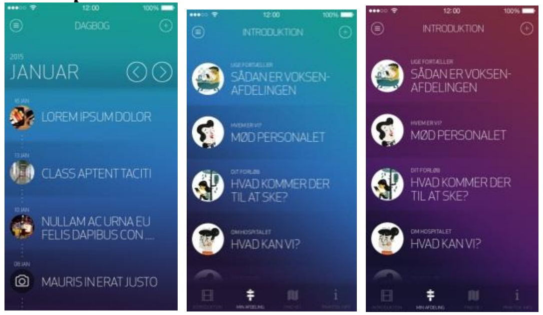<br><br>***Mock-up 4-5***<br>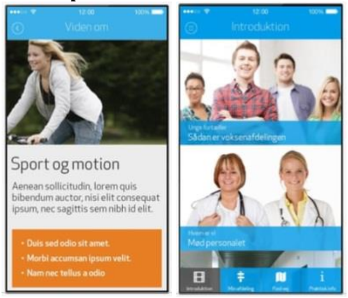 	**Invited**<br>12 young people<br>**Participants**<br>8 young people	**Layout**	**Mock-up 1-3**<br>• Nice colors<br>• Nice interface<br>• Nice, simple, humorous illustrations/icons<br>• Youth-friendly, modern, attractive, interesting<br>• Personal look<br>• Feels "welcoming"<br>• Missing "proximity"<br><br>**Mock-up 4-5**<br>• Nice colors<br>• Nice interface<br>• Like pictures<br>• Better overview<br>• "Proximity"<br>• Hospital-like colors<br>• Needs colors and circles<br>• Totally wrong/boring/ won't notice it<br>• Looks like a leaflet	• Colors and illustrations in mock-up 1-3 were incorporated into the app
**Cycle 2, mail panel**				
Many young people asked for an alcohol lexicon in the app. Such as a place to find the carbohydrates in drinks, like beer, wine etc. What kind of alcohol is important for you to be included in the alcohol lexicon?	**Invited**<br>12 young people<br>**Participants**<br>5 young people	**Content**	• Positive statements about having an alcohol lexicon<br>• Ideas for lexicon content (beer, wine, shots, ciders, drinks)<br>• Include a reminder about "eating before sleeping"	• We included the alcohol lexicon as part of the app.<br>• The lexicon was adjusted according to the suggestions.<br>• A reminder about "eating before sleeping" was added.
**Cycle 3, mail panel**				
Six mock-ups from the app are attached:<br>*The first three illustrate a chat room for young people.*<br>*The last three illustrate a diary function where you can post photos, write your thoughts, and share photos and thoughts with your doctor, nurse or dietician. What do you think when you see the six mock-ups? Primarily in regard to configuration and design.*<br>*Mock-up 1-3*	**Invited**<br>12 young people<br>31 health care providers (12 medical doctors, 14 nurses, 5 dieticians)<br>**Participants**<br>6 health care providers (2 physicians, 3 nurses, 1 dietician)	**Content**<br><br>**Layout**	• Place the positive sides of T1DM up front.<br>• Remember space for "freestyle"<br><br>• User-friendly	• The positive statements were placed in front in Diary and Chat room functions.<br>• We continued with the layout of the Diary and Chat room.

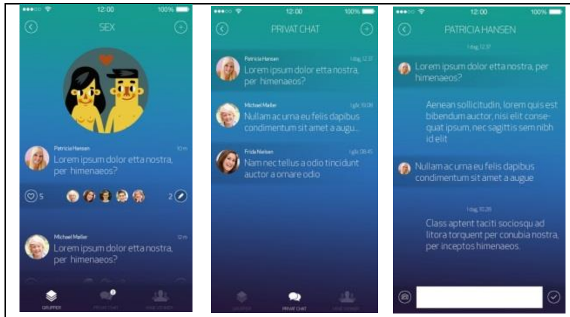<br>*Mock-up 4-6*<br>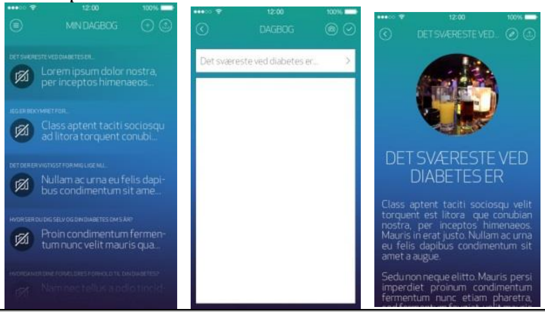			
**Cycle 4, mail panel**			
Two examples of the front page are attached. What are your thoughts about the two options?<br>***Front page 1*** ***Front page 2***<br>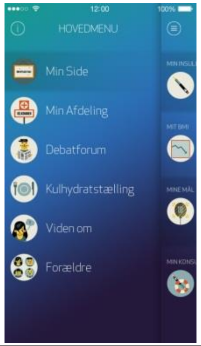<br>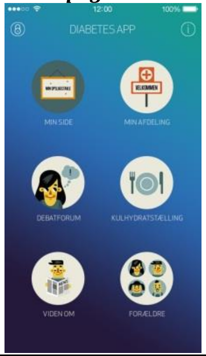	**Invited**<br>23 young people (male = 10)<br>37 health care providers (15 physicians, 16 nurses, 6 dieticians)<br>**Participants**<br>14 young people (male = 6)<br>24 health care providers (11 physicians, 8 nurses, 5 dieticians)	**Layout**<br>**Front page 1**<br>• ”Foreseeable”<br>• Quick overview<br>• Easy to navigate from<br>• Recognizable from other known systems<br>**Front page 2**<br>• Illustrations in focus<br>• ”Foreseeable”<br>• Inviting<br>• Cool icons and colors<br>**In general**<br>• Keep it as simple as possible<br>• Call “parents” function something else	• Front page 1 was chosen due to layout difficulties after a 7th main function was added.<br>• We changed the function heading from “Parents” to “To parents.”
**Cycle 5a, mail panel**			
The app “Young with Diabetes” is almost done! You have a chance to influence the last details. Below is a guide to download the app on your iPhone or android phone.<br><br>*We need to know:*<br>*1. What do you think about the app?*<br>*2. What definitely must be changed before it is finished?*<br><br>*(Test flight attached)*	**Invited**<br>23 young people<br>11 parents<br>38 health care providers (15 physicians, 17 nurses, 6 dieticians)<br>**Participants**<br>4 young people<br>1 parents<br>7 health care providers (4 physicians, 2 nurses,	**Navigation**<br>• Some headings and icons are not intuitive.<br>• It is not clear where to go from the start page (the start page is “My page”).<br>• Risk of log-out when pushing the return button.	• Headings were renamed and icons modified<br>• The start page was changed from being “My Page” to an overview of all major app functions.<br>• Technicalities were fixed
		**Content**<br>• Missed motivating sentences in regard to “my goals.”<br>• Reported missing information, links, and illustrations in the information section<br>• Incorrect information and misspelling was reported	• Motivating sentences were added to “My Goals.”<br>• Information, links, and illustrations were added.<br>• Information and spelling corrected.
		**Layout**<br>• Participants had trouble reading the text because it was small and the colors lacked contrast.<br>• Too many options in the drop-down menu at “my page” and in predefined groups in chat room.	• The text was enlarged and given more contrast.<br>• Removed redundant drop-down menu options and predefined groups to give users a better overview.<br>• Front page at Carbohydrate Counting was adjusted; text

	1 dietician)	• Front page of Carbohydrate Counting was described as confusing with a mix of text in different fonts.	was removed and icons added.
**Cycle 5b, think aloud test**			
Think-aloud scenarios – see Multimedia Appendix 3	**Participants**<br>4 young people<br>3 parents<br>4 health care providers (1 physician, 2 nurses, 1 dietician)	**Navigation**<br>• There was a confusion about the meaning of some of the subheadings so the users misunderstood the function:<br>1. ”Title” on ”My Page”: a provider wrote her profession instead of the intended theme of the note/message<br>2. The “Share with” button: the young people did not dare to push the button because they did not know who they were sharing their thoughts with.<br>• Some headings or icons were not intuitive and users were not able to locate features or functions such as tips packages that were incorporated in the reminder function.<br>• The participants misunderstood an illustration of their quiz score. They thought the system was downloading and waited.<br>• It was not clear where to go from the start page.<br>• Participants were unable to move to the next level when pushing the icon for “share with your provider” (had to push the text)	• Subheadings were modified:<br>1. “Title” was changed to “Theme”<br>2. “Share with” button was changed to “Share with your provider” button<br>• Headings were renamed and icons modified. Tips packages was removed from the reminder function and changed to a major app function.<br>• The “quiz score” illustration was removed and replaced by text.<br>• The start page was changed from being “My Page” to the overview of all major app functions.<br>• The “share with your provider” button was modified so the next level appeared when pushing either the icon or the text.
		**Content**<br>• Participant missed information about food that did not contain carbohydrates.<br>• Unable to download carbohydrate-counting applications referred to in the app	• Information about food that does not contain carbohydrates was added.<br>• Link to application download was added.
		**Layout**<br>• Participants did not discover features such as the option to choose an unfinished sentence when writing a note in the diary or change the notification interval in tips packages.<br>• Participants were unable to see the provider to whom they had sent their message because of the configuration of the page.<br>• Front page at Carbohydrate counting was described as boring with too much text	• Text was added in “Theme” box of the diary: “Select one of the below sentences.”<br>• Text was added to the front page of tips packages describing the possibility of changing the interval of tips notifications.<br>• Technical problems were fixed<br>• Carbohydrate counting front page was adjusted. Text was removed and icons added.
**Cycle 6a, mail panel**			
If you have time, it is still possible to give the last feedback to the app “Young with Diabetes”. We need to know:<br>*1. What do you think about the newest version?*<br>*2. What do you think about the app icon?*<br><br>*(Test flight attached)*	**Invited**<br>23 young people<br>11 parents<br>38 health care providers (15 physicians, 17 nurses, 6 dieticians)<br>**Participants**<br>4 young people<br>2 parents<br>6 health care providers (4 physicians, 1 nurse, 1 dietician)	**Navigation**<br>• Participant felt that the app logged out quickly.	• No changes possible to quick log out since this was a security regulation
		**Content**<br>• Providers reported missing information and missing motivating sentences.<br>• Incorrect information and misspelling was reported.<br>• Illogical sequence of sentences or sections were reported.	• Information and sentences were added.<br>• Information and spelling was corrected.<br>• The text sequences were modified.
		**Layout**<br>• Missing an overview of the long text sections	• The long text sections were split in subsections with subsequent icons and headings. Pushing an icon would bring the reader to a sub-section.
**Cycle 6b, think aloud test**			
Think-aloud scenarios	**Participants**<br>2 young people (male = 1)<br>1 parent (male = 1)<br>2 health care providers (1 nurse, 1 dietician)	**Navigation**<br>• Carbohydrate counting quiz heading was not intuitive.<br>• It was not clear to the users how to adjust the tips package interval.	• Quiz heading was changed to make the quiz more obvious.<br>• A text guide was added at the front page for tips packages.<br>• The options for adjusting the tips package interval were simplified by removing minimum and maximum opportunities and adding the possibility to choose

				between a tip each day or every 2nd day, etc.
		**Content**	• Providers reported missing information.<br>• Incorrect information and misspelling was reported.<br>• Users had difficulty understanding the tips for carbohydrate counting.	• Information was added.<br>• Information and spelling were corrected<br>• The carbohydrate counting tips were modified to make them more understandable.
		**Layout**	• Participants had trouble getting an overview of the name of the chat message.	• The headline of the chat message was enlarged and the name of the chat author was minimized.
**Cycle 7, Feasibility study**				
Evaluation of Feasibility Study	**Participants**<br>6 young people<br>38 health care providers (15 physicians, 17 nurses, 6 dieticians)	**Navigation**	• There was confusion among providers about the web-based mailbox function named "Consultation."<br>• Missing explanation of login page to webmail.<br>• Young people were unable to see if peers or providers replied to their messages unless they logged into the app.	• The web-based mailbox function "Consultation" was renamed "Messages."<br>• Explanations such as "Mail" and "Password" were added to the login box.<br>• Notification features were added so the users received a notification immediately when peers and providers replied.
		**Content**	• Missing information about the background and designers of the app.<br>• Users did not know which providers replied to their message.<br>• Providers reported missing information.<br>• Misspelling was reported.<br>• "Threatening tips about late-term complications"<br>• Young people requested the possibility of searching for peers (by gender, city, age, etc.)<br>• Providers forgot to push the "save" button when setting or canceling an auto-reply at the web-based mailbox.<br>• Providers were unable to search their web-based mailbox for contacts because the search requested an app id number and not a name.<br>• Providers felt that the long list of incoming mails was inconvenient.	• Information about the app was added to an information page.<br>• The name of the provider was added at the top of the message received.<br>• Missing information was added.<br>• Spelling was corrected.<br>• More sensitive wording was used and a few tips were deleted.<br>• Because of limited economic resources, it was not possible to add a "search for peers" button.<br>• The "save" bottom was removed.<br>• The search term was changed from app id number to name.<br>• A delete button was added so the providers could delete messages.
		**Layout**	• Participants did not act on the drop-down menus at "My Page" and the "Reminder" section because the colors lacked contrast.	• Drop-down menus colors were changed to enhance contrast.
