# Supplementary material for: Designing a Self-Management App for Young People With Type 1 Diabetes: Methodological Challenges, Experiences, and Recommendations
Source: JMIR Mhealth Uhealth. 2017 Oct 23;5(10):e124. doi: 10.2196/mhealth.8137 (PMC5673883; doi:10.2196/mhealth.8137)
Supplement: Multimedia Appendix 3 [file mhealth_v5i10e124_app3.pdf]

Multimedia Appendix 3. Think-aloud tasks

Functions	Scenarios, Young People	Scenarios, Parents	Scenarios, Health care providers
**Reminder**	**1.** Set a reminder for your next appointment at the diabetes clinic.<br>**2.** Set a reminder for measuring blood glucose every day at 12pm.<br>**3.** You would like to receive a tip about sport and diabetes. Activate a “sport” tip package.	**1.** Set a reminder for your child’s next appointment at the eye clinic.<br>**2.** Set a reminder for measuring blood glucose every day at 12pm.<br>**3.** You would like to receive a tip about what parents can do to support their teen. Activate a “what parents can do” tip package.	**1.** Help a young patient set a reminder for the next appointment at the diabetes clinic.<br>**2.** Help a young patient set a reminder for measuring blood glucose every day at 12pm.<br>**3.** Help a young patient to receive a daily tip about food. Activate a “food” tip package.
**Information/ Tip Packages**	**4.** You are interested in others’ experiences with having hypoglycemia. Find a video self-portrait of a young person telling about his experiences with hypoglycemia.<br>**5.** You are soon going to take a driver’s license test. Find all the information about driver’s licenses.	**4.** You are interested in others’ experiences with alcohol and T1DM. Find a video self-portrait of a young person talking about alcohol.<br>**5.** Your teen is soon going to take a driver’s license test. Find all the information about driver’s licenses.	**4.** You are interested in young people’s experiences with having hypoglycemia. Find a video self-portraits of a young person telling about his experiences with hypoglycemia.<br>**5.** At a clinic visit, a young patient wants to talk about sex and T1DM. Find all the information about sex and T1DM.
**Chat Room**	**6.** Post a comment to the chat room about hyperglycemia.<br>**7.** Post a party invitation in the chat room.	-	-
**My Department**	**8.** You are going to transfer to the adult diabetes department. Find the phone number needed to book a clinic visit.<br>**9.** You would like to know a little more about your new adult diabetes department. Go watch a movie about the clinic.<br>**10.** You are going to move to the adult department and are told the name of your new physician. Go find information about him.	**6.** Your teen is going to transfer to the adult diabetes department. Go find information about the new diabetes team.<br>**7.** You would like to know a little more about the new adult diabetes department. Go watch a movie about the clinic.	**6.** You are transferring a young patient to the adult diabetes department. You want to show this person his/her new contact physician. Find him in the diabetes-team.<br>**7.** You need to know how things work at the adult department. Go watch a movie about the clinic.
**My Page**	**11.** Your insulin dosage has been changed. Create a note about it at ”My Page.”<br>**12.** At your next consultation, you would like to talk about alcohol and T1DM. Send a message to your health care provider so she is prepared.	**8.** Your teen’s insulin dosage has been changed. Make a note about it at ”My Page.”<br>**9.** At the next consultation, your teen would like to talk about alcohol and T1DM. Help him/her to send a message to the health care provider, so she is prepared.	**8.** You are going to show a young patient how to note his/her goals for next visit. Go and note a goal at “My page”.<br>**9.** You ask your young patient to write to you what he wants to talk about to next consultation. Show him how to do it.
**Carbohydrate Counting**	**13.** You want to improve your carbohydrate counting skills and need to know how the app “Carbs’n Calls” works. Go find a guide.<br>**14.** Start the carbohydrate counting quiz.	**10.** You want to improve your carbohydrate counting skills so you can help your teen. You need to know how the app “Carbs’n Calls” works. Go find a guide.<br>**11.** Start the carbohydrate counting quiz.	**10.** A young patient who is visiting you at the clinic finds it difficult to count carbohydrates. You want to show her the guide to the app Carbs’n Calls. Go find the guide.<br>**11.** You need to know how the carbohydrate counting quiz works. Start the quiz.
